# Biomedical Hydrogels Based on Oxidized Hyaluronic Acid and Carboxymethyl Chitosan Coordinated with Magnesium Ions

**DOI:** 10.3390/biomimetics11090639

**Published:** 2026-09-06

**Authors:** Lei Nie, Yingying Liang, Yiran Lin, Wei Guo

**Affiliations:** College of Life Sciences, Xinyang Normal University, Xinyang 464000, China

**Keywords:** multifunctional dynamic hydrogel, oxidized hyaluronic acid, carboxymethyl chitosan, magnesium ions, hemostatic biomaterial

## Abstract

Rapid hemostasis, oxidative stress resistance, and minimally invasive administration are crucial performance requirements for high-performance wound covering. Inspired by the dynamic remodeling properties of the native extracellular matrix, we fabricated a multifunctional injectable hydrogel through dynamic Schiff-base crosslinking between oxidized hyaluronic acid (OHA) and carboxymethyl chitosan (CMCS), combined with magnesium ion (Mg^2+^) coordination. The effects of Mg^2+^ content on hydrogel properties were systematically investigated. The hydrogels gelled rapidly under physiological conditions and showed good injectability, self-healing behavior, and favorable adhesion to moist tissues. Notably, Mg^2+^ incorporation significantly enhanced hemostatic performance in a mouse tail amputation model, reducing blood loss from 391.7 mg to approximately 75 mg and shortening hemostasis time from 151.7 s to 50.3 s. The 2, 2′-azinobis (3-ethylbenzothiazoline-6-sulfonic acid) (ABTS) radical scavenging efficiency reached approximately 80%, and the hydrogel effectively scavenged intracellular reactive oxygen species (ROS) without compromising cytocompatibility or fibroblast activity. This study presents a biomimetic and easily prepared hydrogel platform that integrates pro-coagulant activity, redox regulation, and on-demand injectability, showing translational potential as bioactive wound covering for bleeding control and oxidative microenvironment regulation.

## 1. Introduction

Chronic non-healing wounds, such as diabetic foot ulcers and pressure injuries, often persist due to sustained oxidative stress, infection, and impaired angiogenesis. They impose a high global prevalence and substantial healthcare burden [1,2]. However, currently available dressings offer limited functionality. They mainly act as physical barriers and absorb exudate, but lack the ability to actively regulate the complex wound microenvironment. Treatment failure is common. For example, some pressure ulcer patients received negative-pressure wound therapy for over a year, yet residual sponge fragments in the granulation tissue caused persistent infection and delayed healing, eventually leading to death [3]. In another case, a diabetic foot ulcer patient underwent debridement and antibiotic therapy, but the wound remained unhealed for 130 days until a combination of advanced dressings was applied [4]. An ideal wound covering should be able to achieve rapid hemostasis, provide reliable wet-tissue adhesion, offer injectability and self-healing, scavenge excess reactive oxygen species (ROS), and maintain a moist healing environment. These functions would help break the vicious cycle of inflammation and infection, and promote the transition from the inflammatory phase to the repair phase [5,6,7].

Hydrogels with excellent injectability can not only load various therapeutic factors, but also fully fill irregular wounds to achieve effective adhesion [8,9]. Meanwhile, self-healing hydrogels can repair structural damage caused by external forces, extending the service life and stability of dressings [10]. Hydrogels are commonly prepared based on natural or synthetic polymers. For instance, carboxymethyl chitosan (CMCS) and hyaluronic acid (HA) are widely used polysaccharides due to their good biocompatibility. HA can be oxidized via periodate to acquire aldehyde-functionalized oxidized hyaluronic acid (OHA). The aldehyde groups of OHA can react with amino compounds to form dynamic Schiff-base bonds. This makes hydrogels possess in situ gelation, self-healing, and shear-thinning behavior. Meanwhile, HA retains its biological advantages, including moisture retention, anti-inflammatory activity, and promotion of cell migration [11,12,13,14]. CMCS is a derivative of chitosan obtained through carboxymethylation. Its molecular chain retains both free amino groups (-NH_2_) and carboxyl groups (-COOH). This improves the poor solubility of native chitosan under neutral or alkaline conditions [15,16]. In addition, its inherent positive charge density, hemostatic properties, and intrinsic antibacterial activity make CMCS particularly suitable as a wound-dressing matrix [17,18]. The OHA/CMCS-based hydrogels exhibited rapid in situ gelation, adhesion to moist tissues, and biocompatibility, showing promise as deep trauma dressings [19].

Wound injury produces excessive ROS that trigger oxidative stress and inflammation, hindering repair. An ideal hemostatic dressing should thus combine rapid hemostasis with ROS scavenging to regulate the redox microenvironment and promote wound healing [20,21]. However, OHA/CMCS Schiff base hydrogels lack the ionic bioactivity to regulate the wound redox microenvironment and promote hemostasis [22]. Magnesium ion (Mg^2+^) serves not only as an essential cofactor for many enzymes but also as an antioxidant that reduces ROS levels and participates in the coagulation cascade to assist hemostasis [23]. In recent years, researchers have successfully incorporated Mg^2+^ into natural polymer hydrogels to improve ROS regulation. Yang et al. developed a photo-crosslinkable dual-network hydrogel based on gelatin methacryloyl (GelMA) and oxidized dextran (ODex), and revealed that the incorporation of Mg^2+^-releasing metal–organic framework (MOF) combined with osteogenic growth peptide (OGP) effectively eliminates excessive ROS via sustained ion release, promotes angiogenesis, and synergistically accelerates bone regeneration in tooth extraction sockets [24]. Dai et al. developed Mg^2+^-enhanced alginate-based ionically crosslinked hydrogels and found that Mg^2+^ accelerated diabetic wound re-epithelialization in vivo [25]. Therefore, designing hydrogel dressings that combine rapid hemostasis with ROS-scavenging capacity remains crucial.

**Scheme 1:**
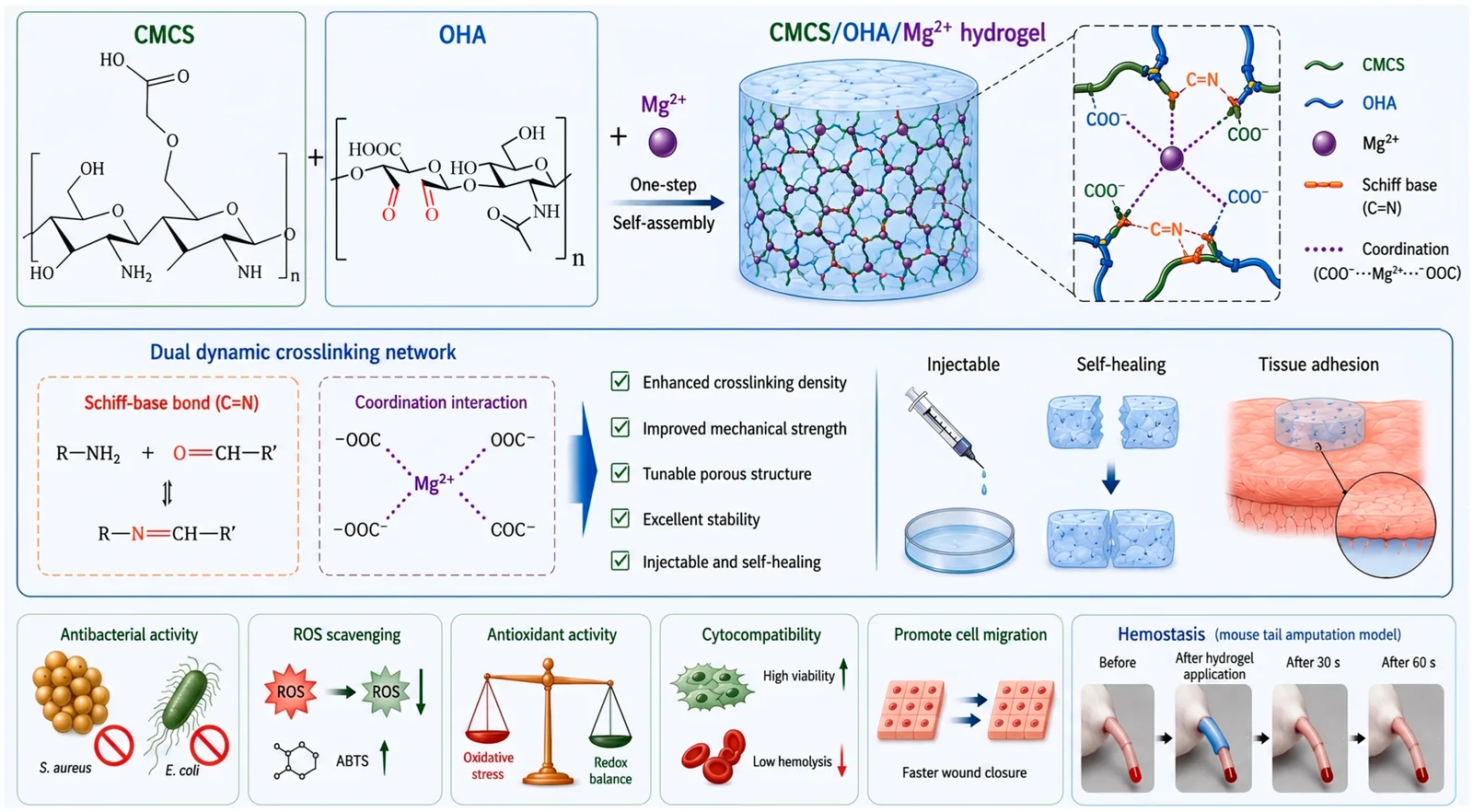
Schematic illustration of the CMCS/OHA/Mg^2+^ hydrogel. The hydrogel crosslinks via dynamic Schiff-base bonds and Mg^2+^ coordination, enabling rapid hemostasis, injectability, self-healing, and antioxidant activity while supporting cell viability at the wound bed.

This study adopts a minimalist, carrier-free design strategy that combines the dynamic adaptability of covalent hydrogels with the bioactivity of magnesium ions. Different from the approaches that encapsulate Mg^2+^ within nanoparticles or MOFs, we directly introduced free Mg^2+^ into the OHA/CMCS Schiff-base network, constructing a multifunctional OHA/CMCS/Mg^2+^ hydrogel through synergistic crosslinking via dynamic Schiff-base bonds and Mg^2+^ coordination (Scheme 1). The resulting hydrogel was mainly intended for a bioactive wound covering, combining rapid gelation, adhesion to wet tissues, effective hemostasis and ROS-scavenging functions in one composition. These properties were chiefly examined in vitro, while the hemostatic effect was tested in vivo using a mouse tail amputation model. Though a complete proof-of-concept for specific clinical applications remains to be established, the present results provide a basis for further studies targeting clearly defined clinical problems in the treatment of wounds.

## 2. Materials and Methods

### 2.1. Chemicals

CMCS, sodium periodate (NaIO_4_, 99.5%), magnesium chloride hexahydrate (MgCl_2_·6H_2_O, ≥98%), and potassium dihydrogen phosphate (KH_2_PO_4_, ≥99.5%) were purchased from Macklin Biochemical Co., Ltd. (Shanghai, China). Disodium hydrogen phosphate dodecahydrate (Na_2_HPO_4_·12H_2_O, ≥99%) and potassium chloride (KCl, ≥99.8%) were bought from Sinopharm Chemical Reagent Co., Ltd. (Shanghai, China). Potassium persulfate (K_2_S_2_O_8_, 99.5%) was purchased from Keyuan Biochemical Co., Ltd. (Heze, China). Sodium chloride (NaCl, 99.8%), 2, 2-diphenyl-1-picrylhydrazyl (DPPH), and hyaluronic acid (HA, 97%, 200–400 kDa) were obtained from Shanghai Enyi Chemical Technology Co., Ltd. (Shanghai, China). 2, 2′-azino-bis (3-ethylbenzothiazoline-6-sulfonic acid) diammonium salt (ABTS) was acquired from Aladdin Biochemical Technology Co., Ltd. (Shanghai, China). Ultrapure water was bought from Sichuan ULUPURE Ultrapure Technology Co., Ltd. (Chengdu, China). All chemicals were employed as received, without further purification.

### 2.2. Preparation of OHA

The preparation of OHA was carried out with modifications based on previously published literature [26]. Briefly, 3 g of HA was dissolved in 300 mL of ultrapure water and stirred (500 rpm, 25 °C) for 8 h to obtain a homogeneous solution. In the dark, 1.62 g of NaIO_4_ was freshly dissolved in 15 mL of ultrapure water and then added to the HA solution. The mixture was continuously stirred (400 rpm, 25 °C) for 12 h in the dark. Subsequently, the mixture was dialyzed against ultrapure water using a dialysis membrane with MWCO of 8–14 kDa for 3 days, with ultrapure water replaced 3 times per day. Finally, the mixture was freeze-dried to yield OHA.

### 2.3. Synthesis of CMCS/OHA/Mg^2+^ Hydrogels

For hydrogel preparation, CMCS (1.6 g) and OHA (1.6 g) were each dissolved in 40 mL of ultrapure water to obtain 4% (*w*/*v*) solutions. Meanwhile, MgCl_2_·6H_2_O (0.61 g) was dissolved in 30 mL of ultrapure water to prepare a 2.03% (*w*/*v*) MgCl_2_·6H_2_O solution. The formulation maintained a constant OHA/CMCS stoichiometric ratio (1:1), with Mg^2+^ content as the sole independent variable. Subsequently, the CMCS, OHA, and MgCl_2_·6H_2_O solutions were mixed at volume ratios of 1:1:0, 1:1:1, and 1:1:2. After gentle shaking, gelation occurred within a few seconds. The obtained hydrogels were designated as (CMCS/OHA/Mg^2+^ 0 (COM0), (CMCS/OHA/Mg^2+^ 1 (COM1), and (CMCS/OHA/Mg^2+^ 2 (COM2), respectively (Table 1).

### 2.4. Fourier-Transform Infrared Spectroscopy (FT-IR) Measurements

Fourier-transform infrared spectroscopy (FT-IR, NicoleliS5, Thermo Fisher, Waltham, MA, USA) was employed to identify the functional groups of OHA and COM hydrogels [15]. Approximately 0.01 g of sample was analyzed using the Attenuated Total Reflectance (ATR) model. FT-IR spectra were recorded over 4000–500 cm^−1^ with 32 accumulated scans at 1 cm^−1^ resolution under dry conditions (relative humidity < 50%).

### 2.5. ^1^H Nuclear Magnetic Resonance (^1^H NMR) Analysis

To further verify the successful oxidation of HA, ^1^H nuclear magnetic resonance (^1^H NMR, JNM-ECZ600R/S3, JEOL, Tokyo, Japan) analysis was performed [15]. A dried sample (approximately 10–20 mg) was dissolved using 500 μL of deuterated water (D_2_O) and vortexed until completely dissolved. The solution was then placed into a 5 mm NMR tube, and the liquid level was brought to approximately 4 cm. ^1^H NMR spectra of OHA were obtained via the noesygppr1D pulse sequence, with relaxation delay (D1) set to 2–4 s, 64–128 scans, and 32K data points. The oxidation degree (OD) of HA was quantified by comparing the integral area of the methyl group protons of pristine HA with that of the anomeric proton peaks in the oxidized structure at 4.8–5.1 ppm, as described in Equation (1):

(1) $$OD\;(\%)\;=\frac{I4.8-5.1}{I1.9/3}\;\times \;100\%$$

### 2.6. Scanning Electron Microscopy (SEM) Images

The cold field-emission scanning electron microscope (SEM, Regulus8220, Tokyo, Japan) was used to observe the morphology and microstructure of COM hydrogels [27]. The lyophilized COM hydrogels were cut into 1 × 1 cm^2^ pieces and mounted on an aluminum stub using conductive adhesive. The samples were subsequently sputter-coated with platinum (15 mA, 60 s) to enhance electrical conductivity. SEM images were acquired at an accelerating voltage of 3 kV using a scanning electron microscope. In addition, the pore size distribution of each sample was quantitatively analyzed from SEM images via ImageJ software (Version 1.53). Moreover, energy-dispersive X-ray spectroscopy (EDX) and elemental mapping further evaluated the elemental composition and distribution of the hydrogels.

### 2.7. X-Ray Computed Microtomography (Micro-CT) Characterization

The porosity and pore dimensions of the lyophilized hydrogel three-dimensional structures were investigated using a Micro-CT scanner (NEOSCAN N80, Mechelen, Belgium). The scanning parameters were set as follows: 50 kV voltage, 80 µA current, 1.85 µm image pixel size, 0.15° rotation step, and 8-frame averaging without a filter. The acquired images were reconstructed with a misalignment correction of –10 pixels. Subsequently, three-dimensional visualization of the reconstructed images was performed using dedicated software. To quantify porosity and pore size, object-based analysis was carried out on 10 separate slices within the volume of interest (VOI) for every sample. Values are reported as the average from 10 slices per sample.

### 2.8. Rheological Properties

The flow behavior of COM hydrogels was characterized at 37 °C with a DHR-2 rheometer (TA Instruments, New Castle, DE, USA). The COM hydrogel samples were placed at the center of a 20 mm aluminum plate with a 1 mm gap and sealed prior to testing. Time sweep tests were conducted over 0–800 s at 1 Hz and 1% strain. Strain sweep and frequency sweep tests were performed at 1 Hz with 0.1–1000% strain and at 1% strain over 0.1–100 rad/s, respectively. Shear rate-dependent viscosity was recorded from 0.1 to 100 s^−1^.

### 2.9. Adhesive Performance

Rubber, wood, glass, plastic, and metal were used as substrates to assess COM hydrogel adhesion. Tissue adhesion was further assessed using fresh chicken tissues (heart, liver, spleen, lung, and kidney). Prior to testing, the tissues were immersed in physiological saline and gently dried. The hydrogels were placed on glass slides and brought into contact with the tissues. After several seconds, the slides were inverted to assess the adhesion behavior. The Ethics Committee of Xinyang Normal University approved the experiments involving laboratory animals (Approval number: XFEC-2026-074).

### 2.10. Self-Healing Properties

The self-healing capacity of the hydrogels was evaluated via macroscopic observation and rheological analysis. For macroscopic evaluation, the hydrogel samples were cut in half, with one piece stained using methylene blue for 5 s. The two pieces were then brought into contact and allowed to heal at room temperature for 5 min (COM0) or 2 h (COM1 and COM2). After healing, the samples were gently lifted with tweezers to assess the healing condition, and the self-healing process was photographed. Rheological self-healing properties were further examined via cyclic strain sweep tests after determining the critical strain. Measurements were conducted at 37 °C under alternating strains of 1% and 300% at 1 Hz for 600 s. Cyclic deformation-induced variations in storage (G′) and loss modulus (G″) were monitored to assess the network recovery capacity of the hydrogel.

### 2.11. Swelling Properties Measurement

To determine the swelling ratio, the mass of freeze-dried COM hydrogel samples was measured (*W*_0_ = 0.01 g) and then immersed in phosphate buffered saline (PBS, pH 7.4, adjusted with 1 M NaOH) at 37 °C. Sample weights were recorded at 1 h intervals until swelling equilibrium was reached. The swollen hydrogels were then removed and weighed again (*W*_1_). The swelling ratio of COM hydrogels was obtained using Equation (2):

(2) $$\mathrm{Swelling}\;\mathrm{Ratio}\;(\%)\;=\frac{W_{1}-W_{0}}{W_{0}}\times \;100\%$$

where *W*_0_ and *W*_1_ represent the initial dry weight and the weight at each time point of hydrogels, respectively.

### 2.12. Antibacterial Activity Evaluation

Following a previously reported method [28], Gram-positive *Staphylococcus aureus* (*S. aureus*, ATCC 6538) and Gram-negative *Escherichia coli* (*E. coli*, ATCC 25922) were employed to evaluate the antibacterial activity of composite hydrogels. Log-phase bacteria (1 × 10^6^ CFU/mL) were co-incubated with hydrogels at 37 °C and 180 rpm for 12 h. Then, the optical density of the bacterial suspension at 600 nm (*OD*_600_) of the treated and control groups was measured to quantify bacterial growth inhibition. The antibacterial efficacy was determined via Formula (3) as follows:

(3) $$\mathrm{Antibacterial}\;\mathrm{Rate}\;(\%)\;=\frac{K_{b}-K_{s}}{K_{b}}\;\times \;100\%$$

where *K_b_* and *K_s_* denote the absorbance of blank and sample groups, respectively.

Additionally, the bacterial suspension was pelleted (10,000 rpm, 5 min) and rinsed once with PBS. The resulting pellet was resuspended to a concentration of approximately 1 × 10^8^ CFU/mL. Then, the suspension (100 μL) was mixed with 1 μL of live/dead staining solution (4′,6-diamidino-2-phenylindole/propidium iodide, DMAO/PI) and incubated at 37 °C in the dark for 15 min. Subsequently, the stained suspension (10 μL) was dropped onto a glass slide, coverslipped, and viewed by fluorescence microscopy (Axio Observer, Carl Zeiss Microscopy GmbH, Jena, Germany).

### 2.13. Antioxidant Activity Evaluation

The antioxidant activity of COM hydrogels was evaluated using an ABTS radical scavenging assay [29]. Briefly, ABTS (0.012 g) and K_2_S_2_O_8_ (0.002 g) were dissolved separately in 3 mL of ultrapure water, mixed, and incubated in the dark at 4 °C for 12 h. The resulting ABTS radical solution was diluted to 8% with ultrapure water. Subsequently, hydrogel samples (0.02 g) were incubated in 3 mL of the diluted solution (37 °C, 100 rpm) for 0.5 h in the dark. The absorbance at 734 nm was recorded via a UV–Vis spectrophotometer (T6 New Century, Beijing Purkinje General Instrument Co., Ltd., Beijing, China). The ABTS scavenging activity was then computed using Equation (4):

(4) $$ABTS\;Scavenging\;Rate\;(\%)=\frac{A_{0}-A_{1}}{A_{0}}\times 100\%$$

where *A*_0_ and *A*_1_ denote the absorbance of the blank control and sample, respectively.

### 2.14. Intracellular ROS Levels Evaluation

The intracellular ROS levels in mouse embryonic fibroblasts (NIH 3T3, CRL-1658™, ATCC, Manassas, VA, USA) treated with COM hydrogels were evaluated using a DCFH-DA fluorescent probe. The H_2_O_2_ solution was diluted with DMEM to a final concentration of 100 μM. The experimental group received 1 mL of H_2_O_2_ solution with 10 μL of hydrogel extract. As controls, the positive and blank groups received H_2_O_2_ solution alone and complete medium alone, respectively. All groups were incubated at 37 °C in the dark for 2 h to induce oxidative stress. Serum-free medium containing DCFH-DA (1:1000) was added at 1 mL per dish to fully cover the cell layer. After incubation at 37 °C under dark conditions for 30 min, the cells were washed three times with PBS and imaged using an inverted fluorescence microscope (Axio Observer, Carl Zeiss Microscopy GmbH, Jena, Germany).

### 2.15. Hemocompatibility Assessment

Following a previously reported method [30], the hemocompatibility of the COM hydrogels was evaluated by a hemolysis assay. Fresh mouse blood (1 mL) was collected in ethylenediaminetetraacetic acid (EDTA)-containing anticoagulant tubes. Red blood cells (RBCs) were collected via centrifugation (3000 rpm, 10 min) and rinsed repeatedly with 0.9% saline until the supernatant became clear. The collected RBCs were then resuspended in saline for subsequent experiments. Hydrogel samples (0.002 g) were immersed in 1 mL of normal saline and incubated for 30 min at 37 °C. Subsequently, 20 μL of RBC suspension was added to each tube, and the tubes were incubated at 37 °C for an additional 1 h. Ultrapure water and 0.9% saline, mixed with an RBC suspension, served as the positive and negative controls, respectively. Following incubation, the supernatant absorbance at 540 nm was measured using a microplate reader. The hemolysis rate was calculated according to Equation (5):

(5) $$\mathrm{Hemolysis}\;\mathrm{Ratio}\;(\%)\;=\frac{A_{s}-A_{b}}{A_{t}-A_{b}}\;\times \;100\%$$

where *A_s_*, *A_t_*, and *A_b_* denote the absorbance values of the supernatants from the sample, positive, and negative control groups, respectively.

### 2.16. Hemostatic Performance Evaluation

To evaluate the in vivo hemostatic performance of the hydrogels, a mouse tail amputation model was established [24]. All animal experiments in this work were carried out in accordance with the Guide for the Care and Use of Laboratory Animals (NIH Publication No. 85-23, revised 1996) and approved by the Experimental Animal Ethics Committee of Xinyang Normal University (Approval No. XFEC-2026-074). Kunming mice (male, 6–8 weeks old, 20–30 g) were used. A total of 12 mice were randomly divided into four groups using a random number table, with 3 mice per group. Hemostatic time and blood loss were recorded by two independent observers blinded to the group allocation. No animals were excluded from the analysis. Each mouse was considered an independent biological replicate, and data are presented as mean ± SD (*n* = 3 per group). Prior to the experiment, mice were fasted for 24 h. The animals were anesthetized with 1% pentobarbital sodium (50 mg/kg of body weight) and immobilized after complete anesthesia. Approximately one-third of the tail (6.3 cm from the tip, total length 19 cm) was amputated to induce bleeding. Pre-weighed hydrogel samples (*W*_1_ = 0.5 g) were immediately applied to the bleeding site until complete hemostasis was achieved [31]. Meanwhile, pre-weighed filter paper (*W*_2_) was placed beneath the wound to absorb the blood loss. The hemostatic time was recorded. Subsequently, the weights of the blood-absorbed hydrogel (*W*_3_) and filter paper (*W*_4_) were measured. The total blood loss (*W*) was calculated according to Equation (6):

*W* = (*W*_3_ − *W*_1_) + (*W*_4_ − *W*_2_) (6)

### 2.17. Cytocompatibility Evaluation

The cytocompatibility of COM hydrogels was assessed with NIH 3T3 cells via fluorescence imaging and Cell Counting Kit-8 (CCK-8) assays. NIH 3T3 cells were cultured in DMEM supplemented with 10% fetal bovine serum (FBS) and 1% antibiotics at 37 °C under 5% CO_2_. Log-phase cells (5 × 10^3^ per well) were seeded into 96-well plates and pre-cultured for 24 h until reaching 70–80% confluence. The medium was then replaced with hydrogel extracts, and cultured for 1, 2, and 3 days. Subsequently, 10 μL of CCK-8 reagent was added, and plates were incubated for 2 h in the dark. Cell viability was evaluated by recording the absorbance at 450 nm (*OD*_450_) via a microplate reader. Furthermore, live/dead staining ([Calcein]-AM/PI) was performed and observed using an inverted fluorescence microscope.

### 2.18. Cell Scratch Assay

The ability of COM hydrogels to promote cell migration was evaluated using a scratch-wound assay. A cell-scratch insert was placed into a 6-well plate. NIH 3T3 cells were then seeded at 5 × 10^4^ cells/well and incubated at 37 °C for 24 h to allow cell adhesion and proliferation. After cell confluence reached approximately 90%, the insert was removed, and COM hydrogel extracts were added for co-culture at 0 h, 6 h, and 12 h. Phase-contrast micrographs were obtained using an inverted microscope, and the migration rate was determined with ImageJ software (Version 1.53) according to Equation (7):

(7) $$Migration\;Rate\left(\%\right)=\frac{A_{0}-A_{12}}{A_{0}}\;\times \;100\%$$

where *A_0_* and *A_12_* are the scratch areas at 0 h and 12 h, respectively.

### 2.19. Statistical Analysis

All data were expressed as mean ± SD (standard deviation) from three independent experiments. Statistical analysis was evaluated by one-way ANOVA and LSD test. A *p*-value < 0.05 was considered statistically significant. Statistical significance was indicated as ns (not significant), * *p* < 0.05, ** *p* < 0.01, and *** *p* < 0.001.

## 3. Results and Discussion

### 3.1. Synthesis of OHA

**Figure 1:**
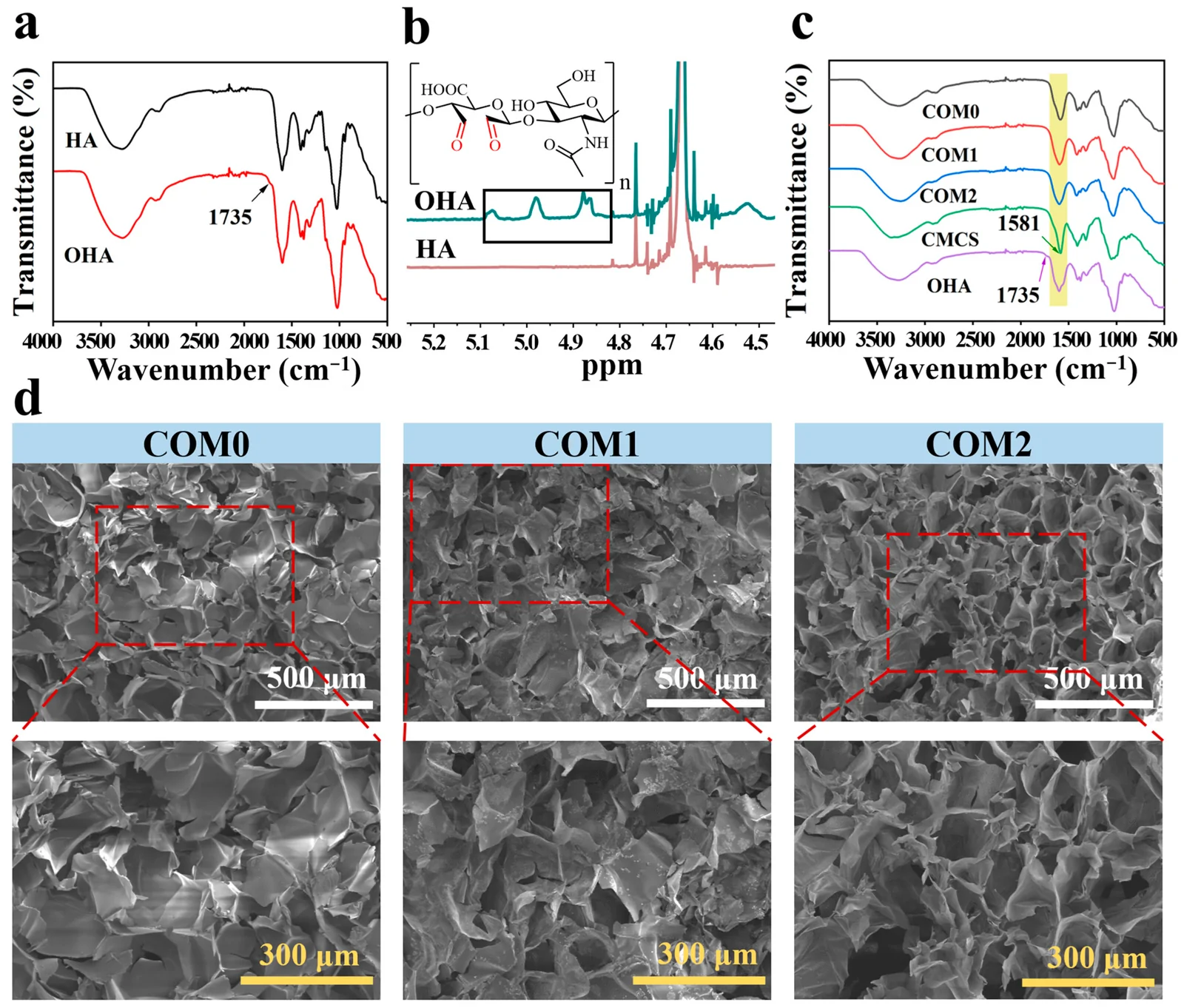
Synthesis and characterization of COM hydrogels. (**a**) FT-IR characterization of HA and OHA. (**b**) ^1^H NMR spectra of HA and OHA. (**c**) FT-IR spectra of COM hydrogels. (**d**) SEM images of COM hydrogels.

The successful preparation of OHA was verified by FT-IR and ^1^H NMR spectroscopy. Compared with native HA, a new absorption peak appeared at approximately 1735 cm^−1^ in the spectrum of OHA (Figure 1a), assigned to the stretching vibration of aldehyde groups. This result indicated that the aldehyde groups were introduced into the HA. In addition, three new characteristic signals appeared at approximately 4.8–5.1 ppm in the ^1^H NMR spectrum, further confirming the successful oxidation of HA to OHA (Figure 1b) [15]. The oxidation degree of OHA was calculated to be 28%, which was consistent with previously reported values [32].

### 3.2. Fabrication of COM Hydrogels

After hydrogel formation, the characteristic aldehyde peak of OHA at 1735 cm^−1^ markedly decreased, which was due to the formation of Schiff-base bonds (C=N) between OHA aldehydes and CMCS amines. Meanwhile, the characteristic carboxyl peak of OHA at 1581 cm^−1^ gradually shifted to higher wavenumbers (1589–1600 cm^−1^) after hydrogel formation and Mg^2+^ incorporation, indicating coordination interactions between Mg^2+^ and the carboxyl groups within the hydrogel network (Figure 1c) [16,33].

### 3.3. Microstructure of COM Hydrogels

**Figure 2:**
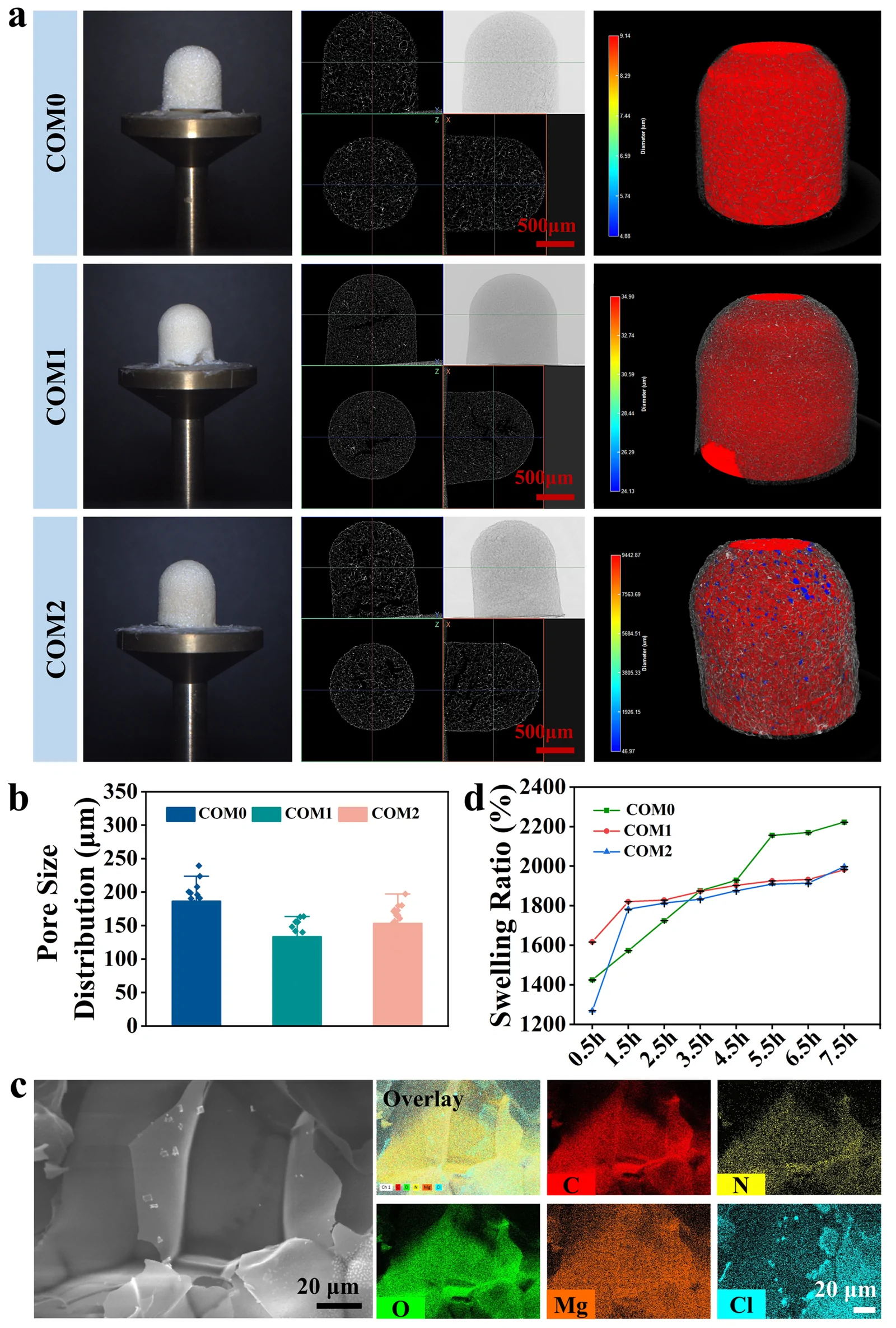
(**a**) Micro-CT characterization of the three dimensional (3D) porous microstructure of COM hydrogels. (**b**) Quantitative pore analysis of COM hydrogel. (**c**) Elemental mappings of COM hydrogel. (**d**) Swelling ratio of COM hydrogels.

The porous three-dimensional architecture of hydrogels facilitates absorption of tissue exudate, maintains a moist wound environment, and supports oxygen transport during wound healing [34]. SEM images showed that all samples exhibited interconnected porous structures (Figure 1d). Pore size analysis using ImageJ (Figure 2b) showed that COM1 exhibited the smallest pore size among the three formulations, while COM2 displayed slightly larger pores than COM1, though both remained smaller than COM0. This non-monotonic trend suggests a dual effect of Mg^2+^ incorporation. At low Mg^2+^ content (COM1), discontinuous ionic crosslinking induced heterogeneous stiffening, where capillary forces drew flexible regions toward rigid domains and compressed the pores during lyophilization. At higher Mg^2+^ content (COM2), the dense, brittle network underwent pore wall rupture and fusion during freeze-drying, leading to pore enlargement. The moderate reduction in polymer concentration from COM0 to COM2 might also contribute to the observed structural differences, though the dominant factor appeared to be the Mg^2+^-mediated ionic coordination [35]. The elemental composition and distribution of the COM hydrogels were further analyzed by EDX mapping (Figure 2c and Figures S1 and S2). C, N, O, Mg, and Cl were uniformly distributed throughout the hydrogel matrix. Notably, the homogeneous distribution of Mg, without obvious aggregation, confirmed the successful incorporation of Mg^2+^ into the hydrogel network. EDX quantitative analysis revealed a gradient in magnesium-ion incorporation (COM0: 0 at.%, COM1: 1.38 at.%, COM2: 2.45 at.%), consistent with the increasing feeding ratio and confirming the tunability of ionic crosslinking density in this system. However, EDX mapping could not exclude sub-micron heterogeneity in ionic crosslink density at the scale of individual crosslink nodes. 

Based on preliminary microstructure analysis using SEM images, Micro-CT was further employed to investigate the 3D microstructure of COM hydrogels [35]. The cross-sectional Micro-CT images from the different (x, y, and z) views clearly showed that the fabricated COM hydrogels possessed a uniform pore size (Figure 2a). Based on a large number of images calculation (360–400 images), the equivalent sizes of COM0, COM1, and COM2 hydrogels were 429.84 ± 41.02 μm, 255.59 ± 28.30 μm, and 321.46 ± 42.51 μm, respectively. In addition, the calculated relative areas (porosities) of COM0, COM1, and COM2 hydrogels were 84.99 ± 0.89%, 77.22 ± 1.39%, and 71.99 ± 2.60%, respectively. Micro-CT directly measures the equivalent diameter of intact interconnected pores through three-dimensional voxel-based reconstruction with a large sample size. SEM, by contrast, provides only two-dimensional cross-sectional chord lengths within a limited field of view [36]. To clearly display pore distribution, we further processed the reconstructed 3D images to mark pores within the hydrogels. In addition, another parameter, average roundness, was derived from Micro-CT images. The average roundnesses of COM0, COM1, and COM2 hydrogels were 0.93 ± 0.02, 0.98 ± 0.02, and 0.84 ± 0.03. The higher average roundness value indicates that shear force has less influence on pore formation and that rare collapse occurs [37]. COM2 hydrogel exhibited the lowest average roundness value, suggesting that the network became globally rigid and brittle, leading to pore wall rupture and coalescence during freeze-drying [38].

### 3.4. Swelling Properties of COM Hydrogels

As water absorption is a critical property of hydrogel dressings for wound recovery, the swelling performance of the COM hydrogels was assessed in PBS [14]. All samples exhibited rapid swelling and reached equilibrium within 7.5 h (Figure 2d). Notably, COM0 showed the highest equilibrium swelling ratio (2222 ± 10.03%), which was consistent with its larger pore size, looser network structure, and higher polymer concentration observed by SEM images.

### 3.5. Rheological Properties of COM Hydrogels

**Figure 3:**
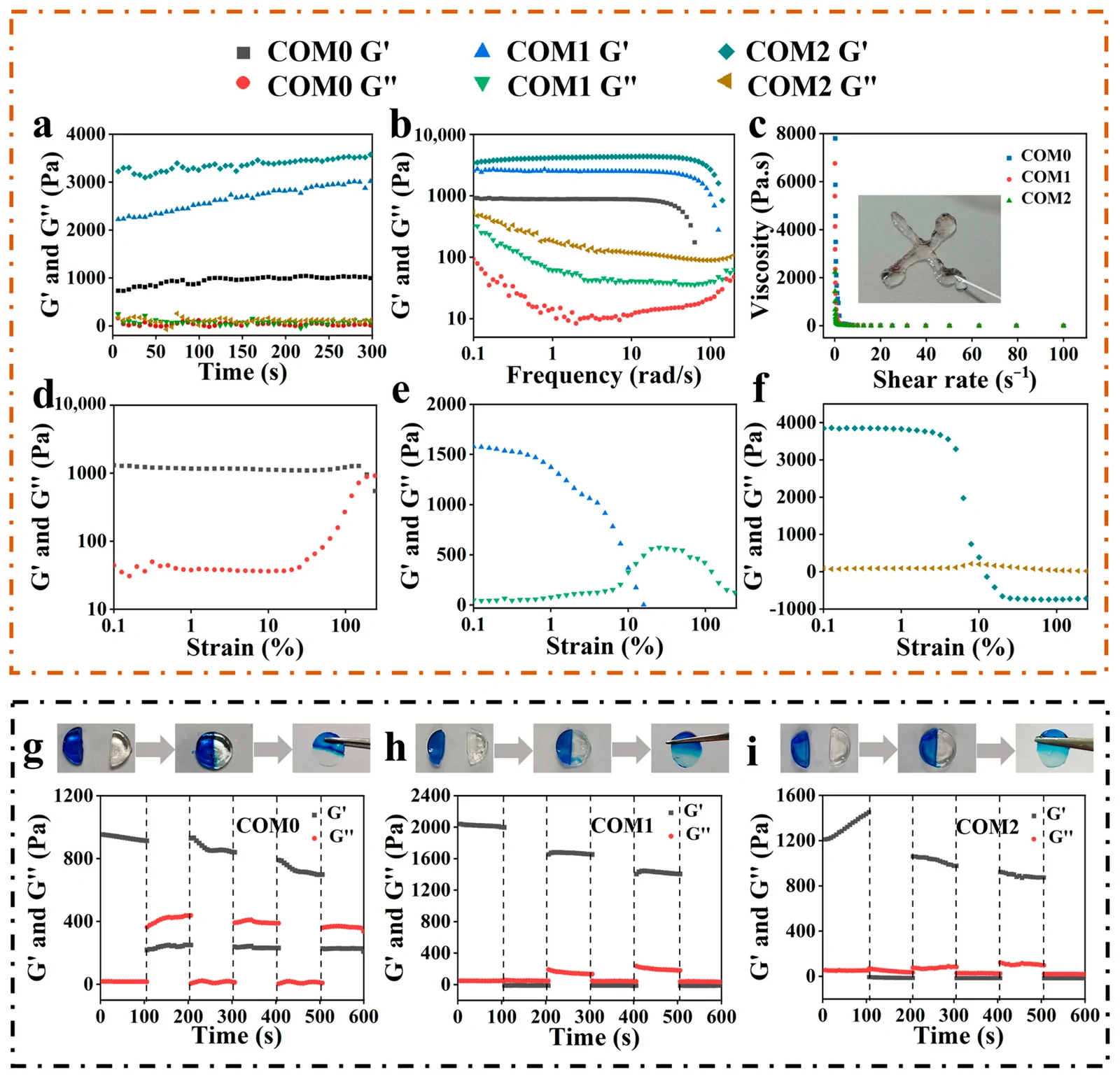
Rheological Properties of COM hydrogels. (**a**) Time sweep, (**b**) Frequency sweep, (**c**) Shear-thinning capacity, and (**d**–**f**) Strain sweep of hydrogels. The inset picture demonstrates the injectability of the prepared hydrogels via a syringe. (**g**–**i**) Step strain measurements for COM0, COM1, and COM2. Photographs showing the self-healing behavior of hydrogels.

Rheology is essential for characterizing the mechanical behavior of hydrogels as biomedical materials. The storage (G′) and loss modulus (G″) of COM hydrogels were displayed as a function of time, strain, frequency, and step strain (Figure 3). G′ consistently exceeded G″ within 300 s, indicating an elastic gel-like behavior (Figure 3a). Notably, the final G′ value surpassed the skin modulus (200–2000 Pa), suggesting that COM hydrogels possessed favorable mechanical strength [39]. Moreover, G′ remained higher than G″ at approximately 100 rad/s (Figure 3b), verifying that these hydrogels displayed viscoelastic solid behavior. COM hydrogel showed shear-thinning behavior under increasing shear. The inset photographs showed that the hydrogels could be extruded through a syringe to form filaments and even write letters (Figure 3c). Based on strain sweep tests, G′ remained higher than G″ within the tested strain range. Above this critical strain, G′ fell below G″ as strain increased, indicating network collapse and fluid-like transition. The collapse strain values of COM0, COM1, and COM2 were approximately 188%, 10%, and 10%, respectively (Figure 3d–f). The presence of Mg^2+^ dramatically reduced the critical strain from 188% to 10%, indicating that ionic crosslinking renders the network more rigid and brittle compared with the purely covalent COM0 network [40]. Notably, no further decrease was observed from COM1 to COM2, suggesting that the network has reached its maximum brittleness at a threshold ionic crosslinking density. The moderate polymer dilution in COM2 may partially counteract the additional stiffening effect from increased Mg^2+^ content. The concomitant reduction in polymer concentration possibly played a secondary role [41]. Furthermore, the negative loss modulus (G″) values observed under high-strain conditions were non-physical artifacts. This phenomenon was attributed to instrumental artifacts caused by edge fracture and sample slippage after network disruption. The self-repairing ability of hydrogel wound dressings allows for prolonged durability and reduces costs. Therefore, the self-healing performance of the hydrogels was investigated. The hydrogels were cut in half and rejoined at 25 °C for 5 min or 2 h. The hydrogels self-healed without external intervention. Moreover, microscopic self-healing was evaluated through rheometer analysis. Rheological tests showed that G′ exceeded G″ at low strain, indicating the formation of the network structure. However, when the strain reached 300%, the collapse of the hydrogel structure was observed, although G′ partially recovered at 1% strain (Figure 3g–i). This was largely attributed to the dynamic Schiff-base bonds between aldehyde and amine groups [42].

### 3.6. Adhesion and Mechanical Properties of COM Hydrogels

**Figure 4:**
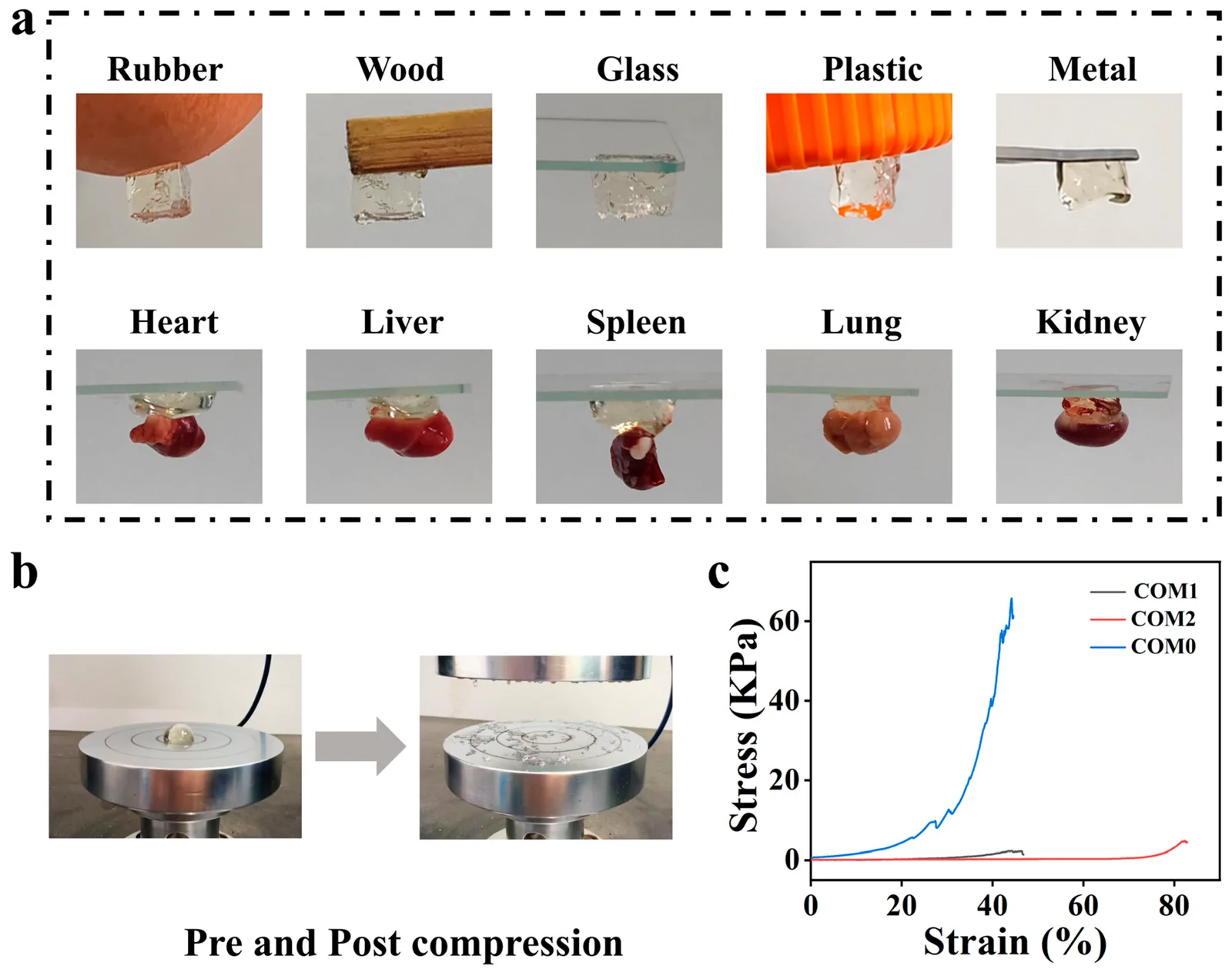
(**a**) Adhesion of COM hydrogels to various surfaces and organ tissues. (**b**) Photos display the COM hydrogels after the compressive stress test. (**c**) Stress–strain curves of COM hydrogels.

Hydrogel adhesion facilitates suturing, tissue repair, regeneration, and wound dressings [43]. The adhesion performance of hydrogels to different substrates was investigated. The hydrogels could adhere to diverse substrates, including rubber, wood, metal, glass, and plastic, as well as biological tissues such as chicken heart, liver, spleen, lung, and kidney (Figure 4a and Figure S3). Compared with conventional bandages, self-adhesive dressings are capable of adhering to the wound site without the need for external support. Hydrogels that possess aldehyde, amino, and carboxyl groups exhibited adhesion to the substrate via a combination of Schiff-base bonds, hydrogen bonds, electrostatic interactions, and van der Waals forces.

The mechanical properties of COM hydrogels were further evaluated through compression tests. All hydrogels fractured after compression (Figure 4b), and their compressive stress–strain curves are shown in Figure 4c. The compressive stress of COM0 reached 65.8 kPa, while that of COM1 and COM2 was only 2.24 kPa and 4.46 kPa, respectively, significantly lower than that of COM0. The marked difference arose from distinct crosslinking mechanisms. Specifically, COM0 relied solely on dynamic Schiff-base crosslinking, which maintained structural integrity under large deformation. Consequently, COM0 accumulated higher compressive stress. In contrast, the introduction of Mg^2+^ in COM1 and COM2 generated additional ionic crosslinking between carboxyl groups of CMCS. This increased crosslinking density but made the network brittle, leading to irreversible failure at small strain and a substantial reduction in compressive strength [44]. COM2 exhibited a greater compressive stress (4.46 kPa) compared to COM1 (2.24 kPa), due to the localized chain aggregation and pore collapse caused by the excess of Mg^2+^, resulting in a heterogeneous but locally denser network which could resist slightly higher compressive loads before breaking, though both were still considerably weaker than the covalent COM0 network [45]. The moderate decrease in polymer concentration from COM0 to COM2 may also have contributed to the lower compressive strength. However, the dominant factor was the change from a single covalent network to a more brittle covalent-ionic dual network [41].

### 3.7. Antibacterial Properties of COM Hydrogels

**Figure 5:**
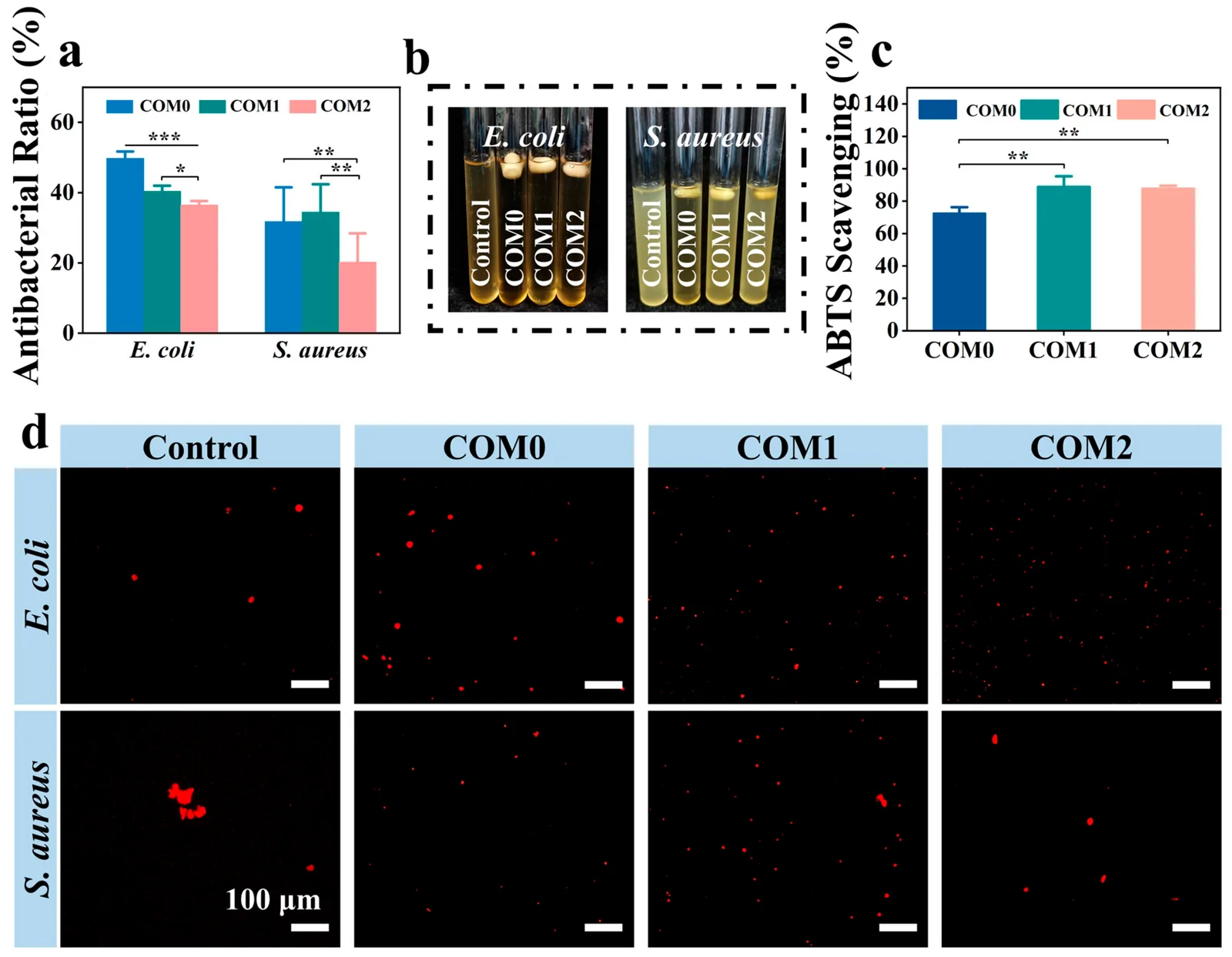
(**a**) Statistical assessment of the antibacterial efficacy against *E. coli* and *S. aureus.* (**b**) Colony growth and hydrogel co-culture images in glass test tubes. (**c**) ABTS scavenging rates of hydrogels. (**d**) Fluorescent images of *E. coli* and *S. aureus* after culturing with COM hydrogels. * *p* < 0.05, ** *p* < 0.01, and *** *p* < 0.001.

Bacterial infection delays tissue healing and causes complications. Accordingly, the antibacterial properties of hydrogels play a key role in modulating the local inflammatory microenvironment [46,47]. Therefore, the antibacterial capacities of COM hydrogels were evaluated. Typical Gram-positive *S. aureus* and Gram-negative *E. coli* were used to evaluate antibacterial properties (Figure 5a,b,d). The hydrogels showed greater antibacterial activity against *E. coli* than *S. aureus*, likely due to their distinct cell wall compositions [48]. While these in vitro results indicate the potential of hydrogels to limit microbial growth, their efficacy against in vivo infection remains to be verified. However, the antibacterial performance of COM hydrogels was limited, possibly because the crosslinking network immobilized CMCS and reduced its release. Future studies will investigate hydrogel degradation in wound microenvironments to release CMCS oligomers and enhance antibacterial performance while maintaining biocompatibility.

### 3.8. Antioxidant and Intracellular ROS Levels Evaluation of COM Hydrogels

Excessive ROS accumulation in wounds exacerbates chronic inflammation and delays healing [49]. The COM hydrogels showed an approximately 80% ABTS free radical scavenging rate (Figure 5c). This was primarily attributed to the hydroxyl and amino groups on the CMCS backbone, which functioned via hydrogen atom and electron transfer pathways [50].

**Figure 6:**
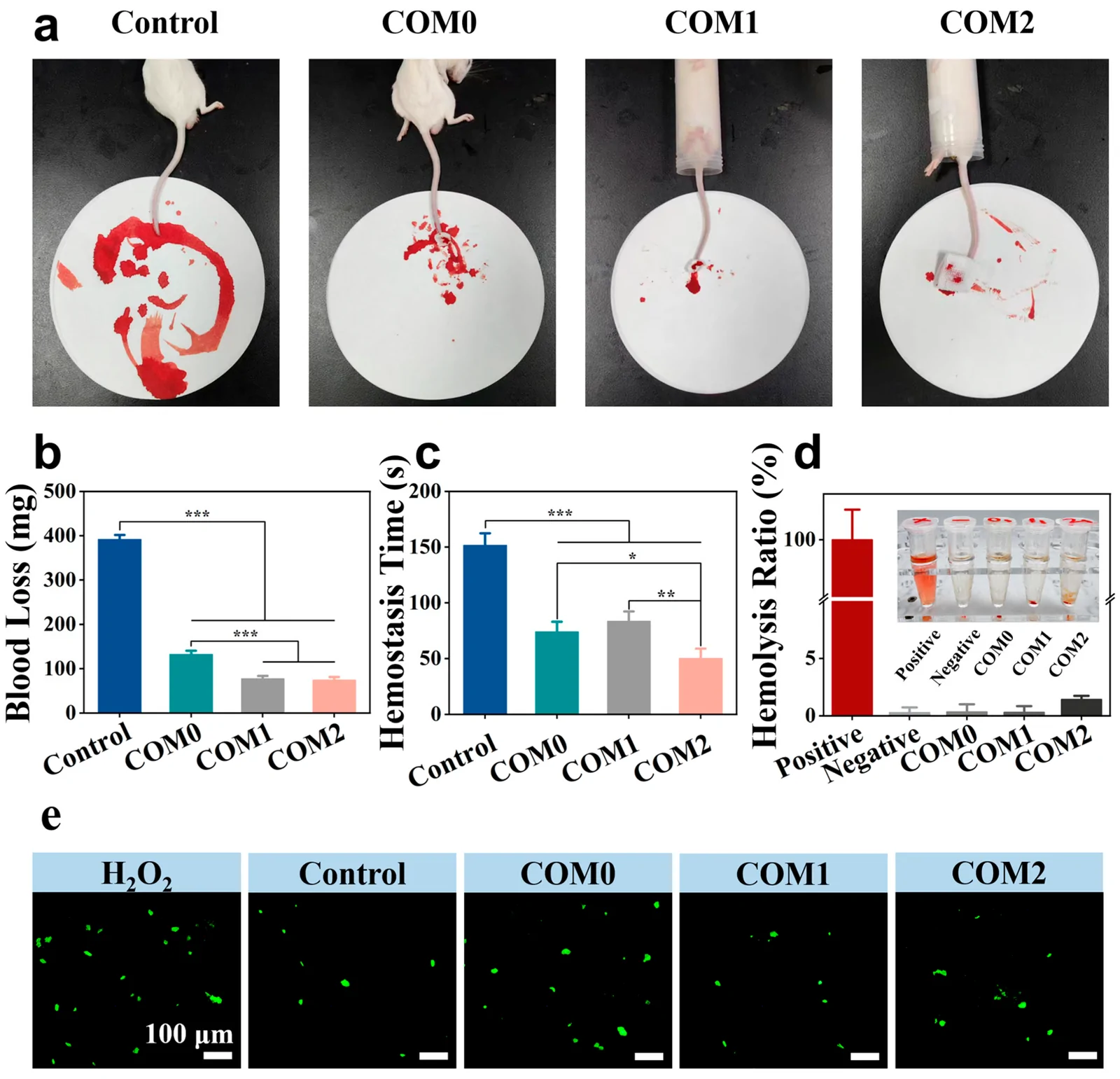
(**a**) Photos of hemostatic progression at the amputated tail treated with hydrogels. (**b**) Blood loss and (**c**) hemostatic time on the mouse tail amputation model. (**d**) Hemolysis and image of COM hydrogels assessed after 1 h co-incubation with fresh mouse blood at 37 °C. (**e**) Fluorescence micrographs of intracellular ROS using NIH 3T3 cells exposed to COM hydrogels. * *p* < 0.05, ** *p* < 0.01, and *** *p* < 0.001.

Furthermore, DCFH-DA staining assessed the protective effect of hydrogels against H_2_O_2_-stimulated ROS in NIH 3T3 cells. Cellular ROS oxidizes nonfluorescent DCFH-DA into fluorescent DCF. Minimal fluorescence was detected in the control group (without H_2_O_2_), whereas pronounced green fluorescence was observed in H_2_O_2_-treated cells, indicating substantial ROS accumulation (Figure 6e). Notably, treatment with the hydrogel extract significantly attenuated the fluorescence signal, with ROS levels similar to those in the control group. These results confirmed the hydrogels’ strong antioxidant activity and their ability to scavenge intracellular ROS effectively.

### 3.9. Hemostatic and Hemolytic Properties of COM Hydrogels

In most cases, bleeding is inhibited through the synergistic effects of vasoconstriction, platelet aggregation, and blood coagulation. However, hemostasis still requires wound dressings, as irregular injuries such as serious artery damage can cause uncontrollable hemorrhage and delay wound healing [51,52,53]. COM hydrogels have flexible shapes, self-healing ability, and favorable adhesion to moist tissues, making them well suited to irregular wounds and effective for hemostasis. Furthermore, the water-absorbing properties of the hydrogels and the role of magnesium ions as cofactors for coagulation factors can endow the condensed hydrogels with accelerated blood-clotting capability. The hemostatic ability of COM hydrogels was investigated using a mouse tail amputation model. The blood diffusion on filter paper was visually assessed, and blood loss and hemostatic time were quantified. Notably, the filter papers in the COM1 and COM2 treatment groups showed minimal blood diffusion (Figure 6a), indicating that COM1 and COM2 could rapidly form blood clots to achieve hemostasis upon contact with the wound. Figure 6b,c showed that average blood loss in the COM1 and COM2 treatment groups was 77.7 and 75 mg, respectively, significantly lower than in the control group (391.7 mg). The COM2 treatment group also significantly shortened the hemostasis time, achieving bleeding cessation within 50.3 s. The hemostasis times in the COM0 (74 s) and COM1 (83.6 s) treatment groups were also markedly lower than in the control group (151.7 s). The results demonstrated that the COM hydrogels had excellent clotting ability and could dramatically shorten the clotting time.

The hemocompatibility of COM hydrogels was assessed via an in vitro hemolysis assay, with hemolysis rates quantitatively determined (Figure 6d). The positive control group exhibited 100% hemolysis, while the negative control group showed 0%. All hydrogel samples displayed hemolysis rates below 5%, meeting the standard specified by the U.S. Food and Drug Administration (FDA). Different from the positive control group, which exhibited obvious hemolysis (bright red solution), the COM hydrogel solutions resembled the negative control, indicating negligible hemolysis. These results demonstrated that COM hydrogels possess favorable blood compatibility, offering critical safety assurance for their biomedical utility.

### 3.10. Cytocompatibility of COM Hydrogels

**Figure 7:**
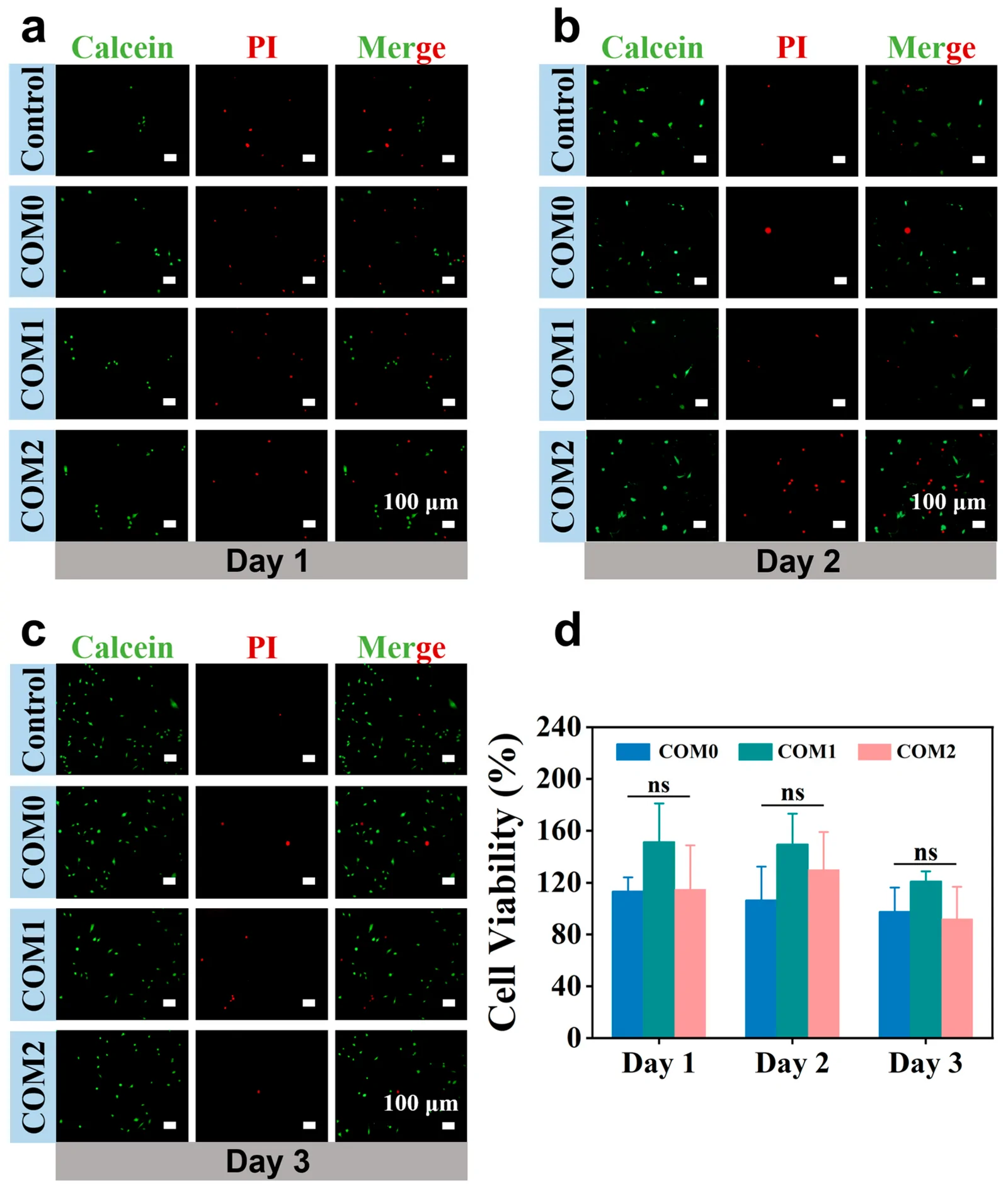
Calcein acetoxymethylester/propidium iodide (Calcein-AM/PI) staining fluorescent images were captured after culturing COM hydrogel extracts with NIH 3T3 cells for 1 (**a**), 2 (**b**), and 3 (**c**) days. (**d**) The cell viability of NIH 3T3 cells cultured with COM hydrogel extracts using the CCK-8 assay. ns, not significant.

Toxicity assessment is essential to the biocompatibility and safety profiling of biomedical materials [54]. First, the cytocompatibility of COM hydrogels was investigated by co-culturing with fibroblasts (NIH 3T3 cells). After exposure to hydrogel extracts for 1, 2, and 3 days, NIH 3T3 cells were stained with live/dead staining (Figure 7a–c). Fluorescence microscopy revealed that cells across all experimental groups primarily exhibited green fluorescence, with an extremely small proportion of non-viable cells emitting red fluorescence. Meanwhile, the adherent fibroblasts exhibited typical spindle morphology with well-extended cytoplasmic protrusions, indicating good adhesion, proliferation, and viability. Furthermore, a CCK-8 assay validated the excellent cytocompatibility of COM hydrogels. After 72 h of co-culture with hydrogel extracts, all sample groups showed good cell viability (Figure 7d), demonstrating that COM hydrogels exhibited excellent cytocompatibility and offered a supportive niche for cell growth.

### 3.11. Cell Migration Assay of COM Hydrogels

**Figure 8:**
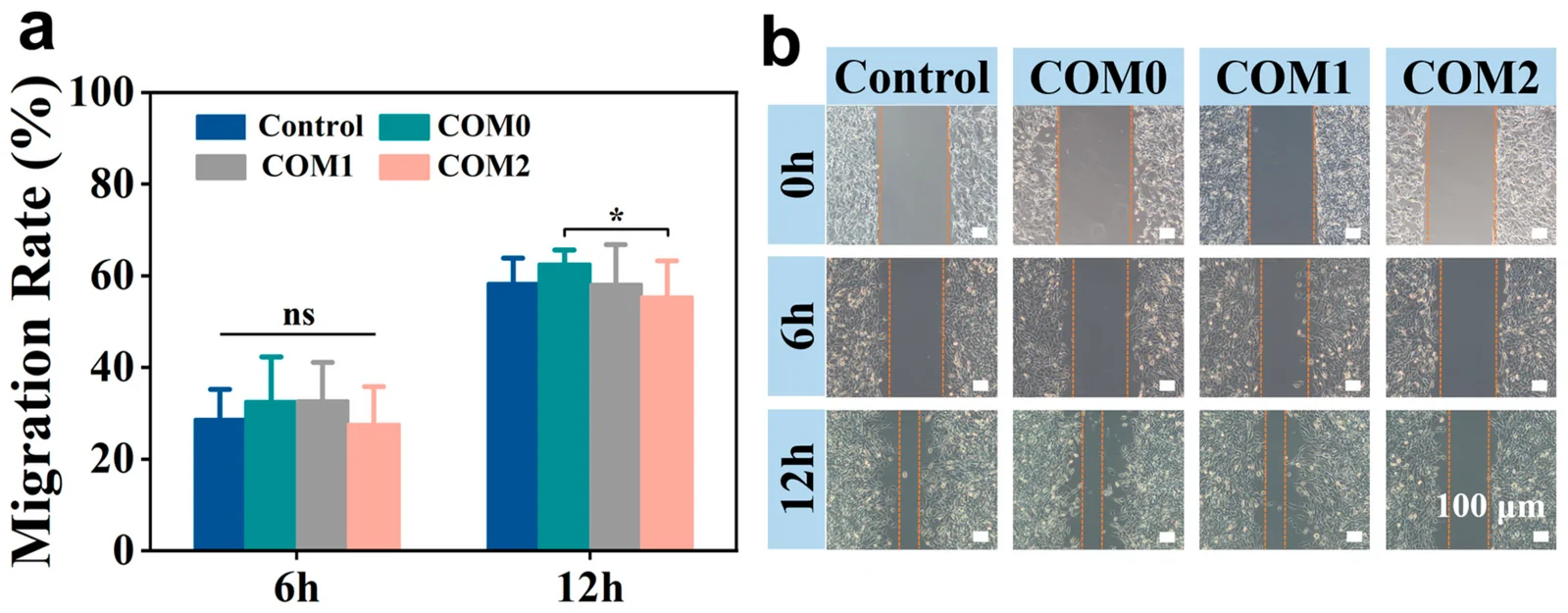
(**a**) Quantification of NIH 3T3 cell migration. (**b**) NIH 3T3 cells scratch assay of different groups for 6 and 12 h. * *p* < 0.05. ns, not significant.

Cell migration was evaluated via an in vitro scratch assay [55]. After co-culture with COM hydrogel extracts for 6 and 12 h, cells in all tested groups showed a cell migration trend (Figure 8b). Quantitative analysis via ImageJ software indicated that the migration rates of the control, COM0, COM1, and COM2 groups at 12 h were 58.43 ± 5.43%, 62.62 ± 2.98%, 58.24 ± 8.52%, and 55.41 ± 7.87%, respectively (Figure 8a). The COM0 group, with the highest polymer concentration, showed a migration rate of 62.62%, slightly above the control (58.43%). This was consistent with literature reports on the good cytocompatibility of CMCS/OHA-based and similar natural polysaccharide systems [56]. The migration rates of COM1 and COM2 decreased slightly (58.24% and 55.41%, respectively) after MgCl_2_ introduction, possibly due to the combined effects of lower polymer concentration and higher ionic strength in the extract medium [57]. CCK-8 results showed no significant cytotoxicity across groups, indicating that the reduced migration was not caused by cell damage. The Mg^2+^-containing hydrogels did not actively promote cell migration, but they also did not impair fibroblast activity, similar to that of many clinically used hemostatic dressings.

## 4. Conclusions

In this study, multifunctional biomedical hydrogels were successfully prepared using OHA and CMCS via dynamic Schiff base bonding, combined with Mg^2+^-mediated coordination interactions. From the perspective of wound management, the developed hydrogel mainly functions as a wound covering. The resulting hydrogel possessed an interconnected porous structure and exhibited excellent injectability, self-healing ability, and wet-tissue adhesion, enabling adaptation to various irregular wound shapes. Mg^2+^ modification significantly enhanced hemostatic performance, reducing bleeding-related complications. In addition, the hydrogel showed favorable antioxidant activity, hemocompatibility, and cytocompatibility, which helped alleviate oxidative stress damage and support cell survival. These properties help break the vicious cycle of infection, inflammation, and delayed healing and promote the transition of chronic wounds from the inflammatory phase to the repair phase. These advantages directly address key clinical challenges in wound care, including uncontrolled bleeding and excessive oxidative stress. The fixed OHA/CMCS stoichiometric ratio and constant total volume result in a moderate decrease in total polymer concentration from COM0 (4%, *w*/*v*) to COM2 (2%, *w*/*v*) as the Mg^2+^ solution volume increases. The concurrent dilution of the polymer may also cause a secondary interference effect. A quantitative study of ion-release behavior should be conducted to further clarify the relationship between structure and bioactivity. This study still lays the foundation for developing injectable hydrogels with combined hemostatic and antioxidant functions, with potential applications in wound care, hemostatic adjuncts, or bioactive dressings. Further studies are needed to evaluate whether this hydrogel’s healing efficiency meets clinical requirements. In particular, investigations of collagen deposition, re-epithelialization, angiogenesis, and macrophage polarization, with a focus on specific wound types and customized designs, will clarify how this system forms a reparative ionic microenvironment and promotes the transition of wounds from the inflammatory stage to the tissue regeneration stage, thus addressing a critical clinical problem.

## Figures and Tables

**Table 1:** The components of CMCS, OHA, and Mg^2+^ for the preparation of COM hydrogels.

Sample	CMCS Solution (mL) ^a^	OHA Solution (mL) ^a^	MgCl_2_·6H_2_O Solution (mL) ^b^	Total (mL)	Polymer Conc. (%, *w*/*v*)	Mg^2+^ Conc. (%, *w*/*v*) ^c^
**COM0**	0.5	0.5	0	1.0	4.0	0
**COM1**	0.33	0.33	0.33	1.0	2.67	0.081
**COM2**	0.25	0.25	0.5	1.0	2.0	0.121

^a^ A total of 4% (*w*/*v*) for CMCS solution and OHA solution. ^b^ A total of 2.03% (*w*/*v*) for MgCl_2_·6H_2_O solution. ^c^ Mg^2+^ concentration (%, *w*/*v*). Conc., concentration.

## Data Availability

Data will be made available on request.

## Supplementary Materials

The following supporting information can be downloaded at: https://www.mdpi.com/article/10.3390/biomimetics11090639/s1, Figure S1: EDX spectrum and elemental composition data of COM1 hydrogel; Figure S2: Elemental mappings of COM0 and COM2 hydrogels were shown in (a) and (c), respectively. The EDX spectra and elemental composition data of COM0 and COM2 hydrogels are displayed in panels (b) and (d), respectively. Figure S3: The adhesive behavior of COM hydrogels on the surface of rubber, wood, glass, plastic, and organ tissues such as heart, liver, spleen, lung, and kidney. (COM1 and COM2 hydrogels).
