# Supplementary material for: Biomedical Hydrogels Based on Oxidized Hyaluronic Acid and Carboxymethyl Chitosan Coordinated with Magnesium Ions
Source: Biomimetics (Basel). 2026 Sep 6;11(9):639. doi: 10.3390/biomimetics11090639 (PMC13604675; doi:10.3390/biomimetics11090639)
Supplement: Supplementary file 1 [file biomimetics-11-00639-s001.zip › biomimetics-4488643-supplementary.pdf]

Supporting Information

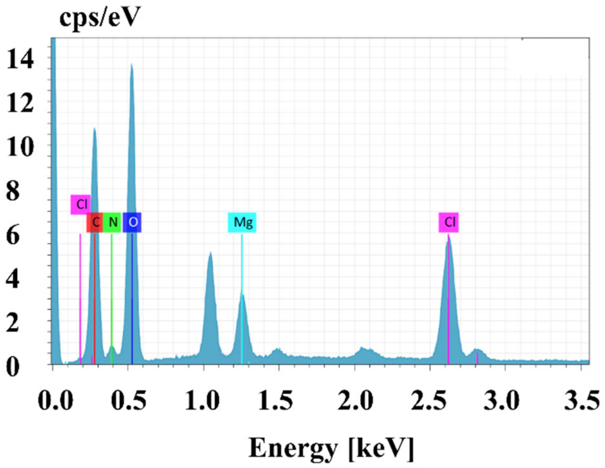

Element	At%	wt%
C	47.24	38.67
N	6.80	6.49
O	41.60	45.36
Mg	1.38	2.28
Cl	2.98	7.20
total	100.00	100.00

Figure S1. EDX spectrum and elemental composition data of COM1 hydrogel.

a

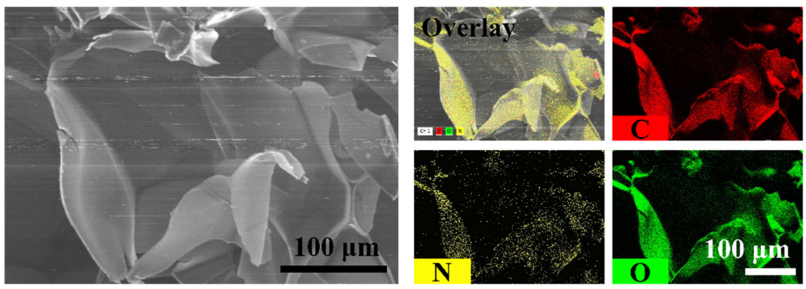

b

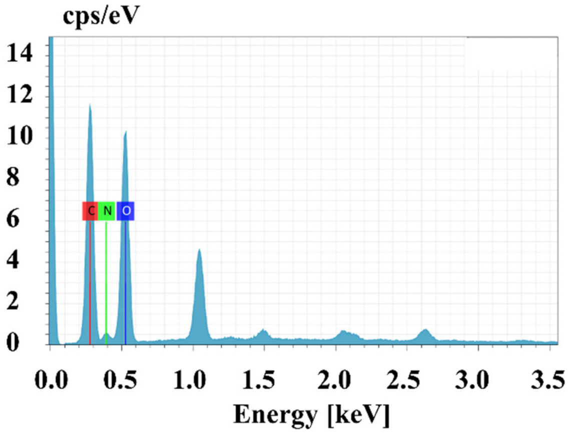

Element	At%	wt%
C	45.98	39.34
N	6.43	6.41
O	47.60	54.25
Mg	0.00	0.00
Cl	0.00	0.00
total	100.00	100.00

c

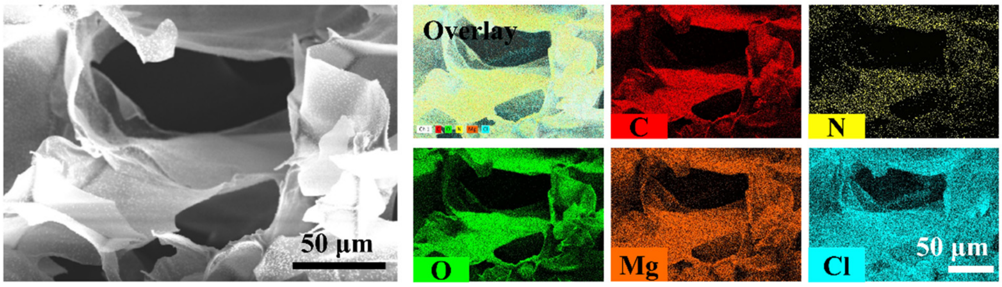

d

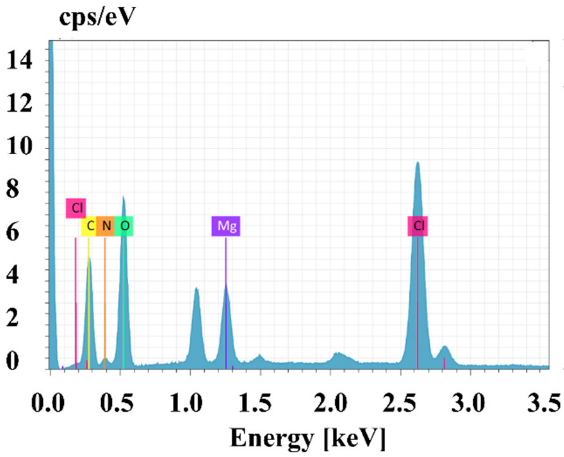

Element	At%	wt%
C	48.65	37.87
N	6.34	5.75
O	35.91	37.23
Mg	2.45	3.86
Cl	6.66	15.29
total	100.00	100.00

Figure S2. Elemental mappings of COM0 and COM2 hydrogels were shown in (a) and (c), respectively. The EDX spectra and elemental composition data of COM0 and COM2 hydrogels are displayed in panels (b) and (d), respectively.

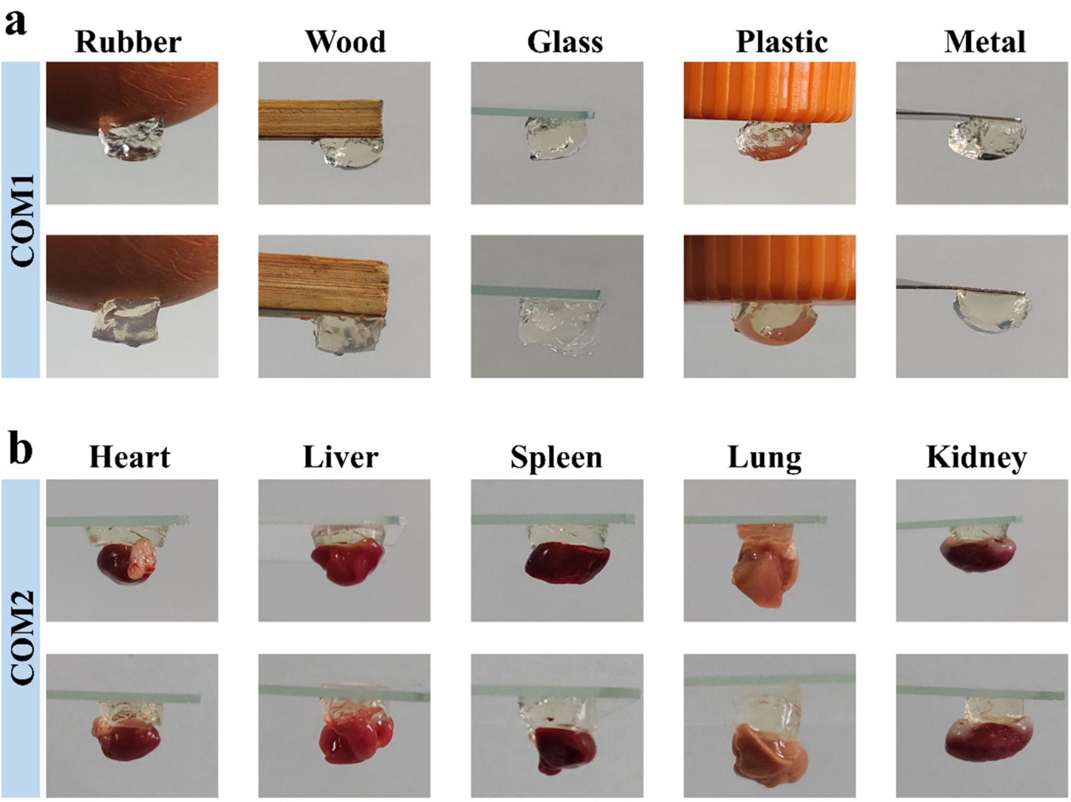

Figure S3. The adhesive behavior of COM hydrogels on the surface of rubber, wood, glass, plastic, and organ tissues such as heart, liver, spleen, lung, and kidney. (COM1 and COM2 hydrogels)
